# Hydrogen Sensor: Detecting Far-Field Scattering of Nano-Blocks (Mg, Ag, and Pd)

**DOI:** 10.3390/s20143831

**Published:** 2020-07-09

**Authors:** Eunso Shin, Young Jin Lee, Hyoungjoo Nam, Soon-Hong Kwon

**Affiliations:** 1 Department of Physics, Chung-Ang University, 84, Heukseok-ro, Dongjak-gu, Seoul 06974, Korea; ensoshin21@gmail.com (E.S.); youngjin.lee.91@gmail.com (Y.J.L.); 2 Da Vinci College of General Education, Chung-Ang University, 84, Heukseok-ro, Dongjak-gu, Seoul 06974, Korea; todayonlybabo@cau.ac.kr

**Keywords:** plasmonic sensor, hydrogen sensor, far-field, scattering

## Abstract

Hydrogen sensor technologies have been rapidly developing. For effective and safe sensing, we proposed a hydrogen sensor composed of magnesium (Mg), silver (Ag), and palladium (Pd) nano-blocks that overcomes the spectral resolution limit. This sensor exploited the properties of Mg and Pd when absorbing hydrogen. Mg became a dielectric material, and the atomic lattice of Pd expanded. These properties led to changes in the plasmonic gap mode between the nano-blocks. Owing to the changing gap mode, the far-field scattering pattern significantly changed with the hydrogen concentration. Thus, sensing the hydrogen concentration was able to be achieved simply by detecting the far-field intensity at a certain angle for incident light with a specific wavelength.

## 1. Introduction

Hydrogen is one of the most promising future energy sources and carriers, owing to its renewability, high efficiency, and zero greenhouse gas emissions [1]. The hydrogen sensor is a crucial device for developing safe hydrogen source technology because hydrogen is flammable and explosive at a concentration >4% in air [2]. In addition, hydrogen gas is colorless and odorless; it consists of the lightest molecule, which means that it leaks easily and is hard to detect. Many types of hydrogen sensors have been developed by exploiting the changes in the material properties due to hydrogen absorption: thermal conductivity, electrochemical, electric resistance, and optical sensors [3,4]. Optical sensors are promising because of their high sensitivity and fast response. In addition, they are unaffected by the electromagnetic environment and provide no source of ignition, such as a spark. The most effective optical hydrogen sensors are based on plasmonic resonance; they detect a shift in the resonant wavelength with respect to the hydrogen concentration [5,6]. However, these methods have a spectral resolution limit because metal absorption causes broadening in the resonance linewidth. In other studies, the linewidth of the resonant wavelength has been made relatively sharper by using the nanogap between palladium (Pd) and gold (Au) nanoparticles; nevertheless, the resonance sensor still has limited sensitivity, owing to the inherent resonance linewidth [7,8]. However, the far-field scattering intensity changes sensitively with the shifting plasmonic resonance of nanostructures [9]. In particular, the far-field scattering of the plasmonic gap mode between two nano-blocks with a subwavelength gap is highly sensitive to the property changes of each block, such as structure size, optical property, and gap distance. In other words, detecting the far-field scattering intensity can go beyond the spectral resolution limitation. To overcome the limited spectral resolution, we used optical hydrogen sensors composed of Pd, Mg, and Ag nano-blocks to detect the far-field scattering of the plasmonic gap mode. The atomic lattice of Pd expands, and the optical constants change after the absorption of hydrogen [10,11,12], which can change the plasmonic gap mode. In addition, metallic Mg becomes a dielectric material (MgH_x_) when it absorbs hydrogen [13]. This Mg property changes the plasmonic gap mode. The far-field scattering intensity changes greatly with the hydrogen concentration, owing to the properties of the materials. In this study, the proposed plasmonic sensors exploited the sensitive changes in the angular distribution of the scattering intensity in two or three metal nano-blocks to detect hydrogen gas, while most plasmonic hydrogen sensors used the resonance shift. Thus, the proposed sensor had no spectral resolution limit due to Ohmic loss, which is unavoidable in most plasmonic resonance sensors. In this study, we combined the optical constant changes of Mg and Pd to design the plasmonic hydrogen sensor, which exhibited a greater sensitivity than a sensor with only one hydrogen reactional metal block.

In [14], a hydrogen sensor composed of Pd–Au nano-dimers was proposed; the ratio of the left- and right-directional scattering intensities changed according to the hydrogen concentration. In [15], the great reflectance that changed because of the enhanced diffused light during hydrogen absorption was exploited. Compared to these two research studies that focused on the directionality of the scattering intensity, the proposed sensor of this study (which combined Pd and Mg nano-blocks) achieved greater scattering intensity changes and a monotonic increase in the angular ratio of the scattering intensity.

The remainder of the manuscript is organized as follows: Section 2 introduces the basic concept of the proposed sensors, including the material properties and structure parameters. In Section 3, the simulation results of three sensors are presented and analyzed based on the far-field scattering intensity spectrum. Finally, Section 4 provides a summary and conclusions.

## 2. Materials and Methods

**Figure 1:**
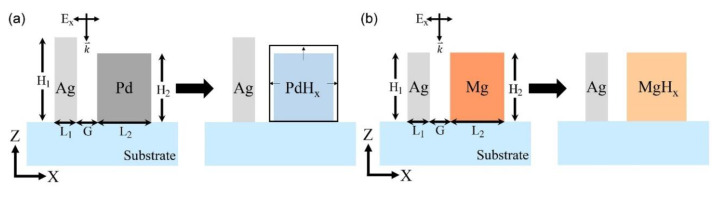
Schematic of hydrogen sensor structures. (**a**) This structure consists of a silver (Ag; H_1_ = 230 nm, L_1_ = 50 nm) and a palladium (Pd) block (H_2_ = 200 nm, L_2_ = 150 nm) with a 50 nm gap (G = 50 nm). When the Pd absorbs hydrogen, the Pd lattice expands. (**b**) This structure consists of an Ag (H_1_ = 200 nm, L_1_ = 50 nm) and a magnesium (Mg) block (H_2_ = 200 nm, L_2_ = 150 nm) with a 50 nm gap (G = 50 nm). When the Mg absorbs hydrogen, it becomes magnesium hydride (MgH_x_), which is a dielectric material.

We proposed sensing methods that can measure hydrogen concentration by using the extraordinary properties of Pd and Mg and detecting the far-field pattern changes at a specific angle. For effective and precise hydrogen sensing, two nano-sensors composed of Ag, Pd, and Mg nano-blocks were proposed, as shown in Figure 1. The two sensors are composed of two metal nano-blocks that have an infinite dimension in the y-direction and a SiO_2_ substrate. The first sensor, which is composed of Ag (200 nm × 150 nm) nano-blocks, can identify the presence or absence of hydrogen gas. The second sensor, which is composed of Ag (230 nm × 50 nm) and Pd (200 nm × 150 nm) nano-blocks, provides detailed information about the hydrogen concentration. Both sensors consist of metal–insulator–metal (MIM) cavity structures of two nano-blocks with 50 nm air gaps.

Palladium (Pd) and Mg have unique properties when they absorb hydrogen. In Pd, the absorbed hydrogen occupies an octahedral site on the face-centered cubic lattice, which results in palladium hydrogen (PdH_x_). In addition to optical constant changes, the Pd block is affected by the volumetric atomic lattice expansion (as shown in Table 1) because of the phase transition from an α to a β phase [16]. When the hydrogen concentration (H/Pd) increases from 0 to 0.82, which means that the hydrogen pressure increases to 8037 Pa, the Pd lattice expands to 3.57% [12]. Owing to this property, the width (L_2_) of the Pd nano-block expands from 150 to 155.35 nm, and the height (H_2_) expands from 200 to 207.14 nm. We exploited these expansion properties to change the MIM cavity between the Ag and Pd blocks with the hydrogen concentration, as shown in Figure 1a. When the Pd lattice expanded isotropically, the gap between the blocks decreased, and the cross-section of the resonance wavelength increased. The increase in the cross-section of the metal blocks dominated over the former effect and caused great changes in the far-field scattering intensity.

When Mg absorbs hydrogen, the metallic Mg begins its transition into a dielectric material (MgH_x_) [17,18]. This Mg transition depends on the hydrogen-to-metal ratio; however, no accurate research results of the optical properties with respect to the hydrogen concentration have been presented. We used the optical properties of MgH_x_ at a hydrogen concentration (H/Mg) of 1.3 ± 0.5 [13]. A sensor composed of Ag (unable to absorb hydrogen) and Mg (can absorb hydrogen) nano-blocks can detect great far-field pattern changes in the presence of hydrogen. The size of the effective gap mode that was originally highly localized between the Ag and Mg nano-blocks changes greatly because an electric (E) field is formed inside the resulting dielectric MgH_x_. With such a great change in the pattern, the presence or absence of hydrogen gas can even be easily determined in a far-field. The process of both hydrogen reactions of Pd and Mg are reversible through dehydrogenation using oxygen.

In this study, the sensing method with the MIM cavity mode and far-field radiation was simulated with COMSOL Multiphysics and the finite-element method. We used a SiO_2_ substrate [19] and light that is linearly polarized and propagates in the x- and z-directions, respectively. The Mg and MgH_x_ material properties (n, k) were based on [13], and those of Ag were based on [20]. In addition, the material properties of Pd and PdH_x_ were based on the Drude model. Table 1 shows the plasma frequency ($\omega_{p}$), dielectric constant ($\varepsilon_{\infty }$), and relaxation time (1/γ).

(1) $$\varepsilon \left(\omega \right)=\varepsilon_{\infty }-\frac{\omega_{p}{}^{2}}{\omega^{2}+i\gamma \omega }$$

For state-of-the-art-fabricated plasmonic devices, the reported surface roughness values of Pd and Ag nanostructures are 0.5 nm [21] and 0.2 nm [22], respectively. The particle sizes on the metal surfaces can be decreased with several elaborate fabrication techniques, such as the deposition parameter control [22], template stripping method [21,23], and thermo-assisted spin coating [24]. With increasing roughness, the Ohmic absorption loss and scattering loss in the metal surfaces increases [25,26]. Therefore, the directional scattering light in plasmonic devices can be degraded, and the sensing performance can be decreased.

## 3. Results

### 3.1. Hydrogen Sensor with Ag and Pd Nano-Blocks

**Figure 2:**
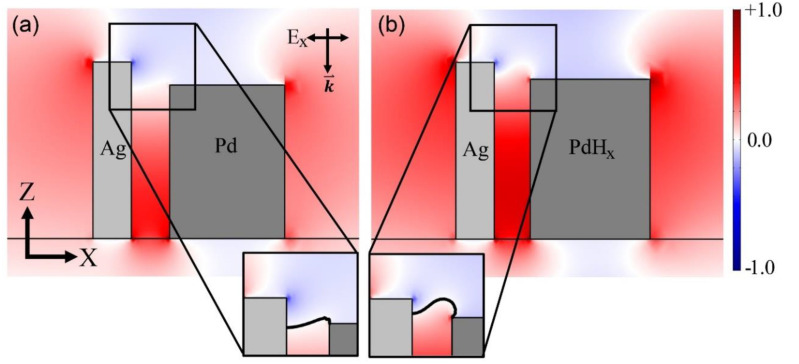
E_x_ field profiles of sensors composed of Ag and Pd nano-blocks for incident light with a wavelength of 1000 nm (**a**) before and (**b**) after hydrogen absorption. (**b**) Pd lattice expansion of 3.57%; the change in the optical constant was investigated for a hydrogen concentration (H/Pd) of 0.82. Insets represent enlarged upper ends of the two blocks. Solid lines in the insets represent the boundary at which the E_x_ field is zero.

The first proposed hydrogen sensor was composed of Ag and Pd nano-blocks. The incident electromagnetic wave propagated in the negative z-direction and was linearly polarized in the x-direction. Figure 2 presents the E_x_ fields of the sensor before and after hydrogen absorption. The plasmonic cavity mode is localized between the Ag and Pd blocks. In addition, the solid lines in the insets represent the zero amplitude of the E_x_ field. By comparing the two figures, it can be noted that the upper end of the plasmonic cavity mode shifts upward when the Pd nano-block absorbs hydrogen (i.e., the solid line in the insets moves upward). This is due to the atomic lattice expansion of Pd when it experiences a phase transition owing to hydrogen absorption. Because the cavity mode changes, the far-field scattering distribution changes.

**Figure 3:**
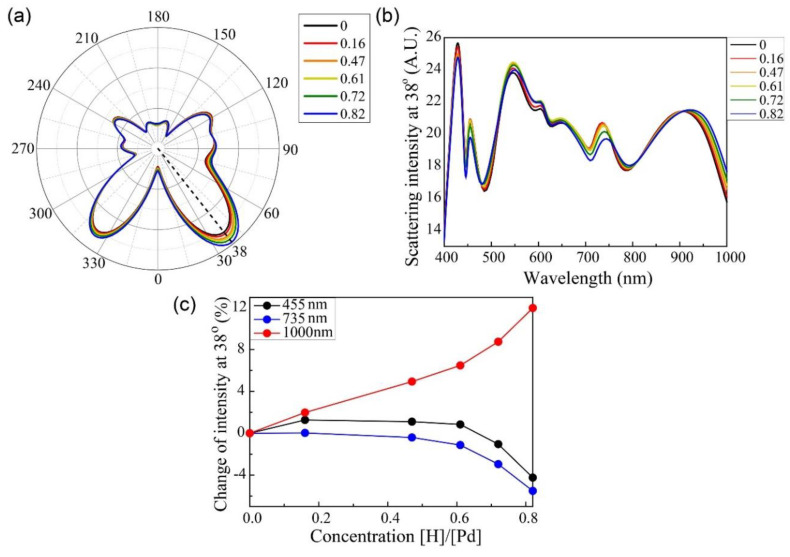
(**a**) Far-field scattering radiation at 1000 nm with respect to hydrogen concentration. (**b**) Far-field scattering at 38° for wavelengths of 400–1000 nm. (**c**) Differences in the far-field scattering intensities at three different wavelengths for hydrogen concentrations of 0–0.82 at 38°.

Figure 3a presents the far-field polar graph with respect to the hydrogen concentration; 0° indicates the direction of the incident light (i.e., “forward scattering”), and 180° indicates the opposite direction (i.e., “backward scattering”). The far-field radiation patterns of the incident light with 1000 nm wavelength changed with the hydrogen concentration, particularly the forward scattering region. In addition, the scattering intensity was significantly changed at 38°; it was proportional to the hydrogen concentration of the Pd nano-block (H/Pd), which increased from 0 to 0.82. The far-field intensities at 38° are plotted as a function of the incident wavelength for different hydrogen concentrations in Figure 3b. At certain specific wavelengths (455, 735, and 1000 nm), the far-field intensities strongly depended on the hydrogen concentration. Figure 3c presents the relative difference (in %) of the far-field scattering intensities at 38° and 455, 735, and 1000 nm wavelengths with respect to the hydrogen concentration. At 455 and 735 nm, the far-field radiation decreased slightly when the hydrogen concentration increased from 0 to 0.82. At 1000 nm, the far-field intensity increased monotonically in the investigated range of the hydrogen concentration. In particular, the scattering intensity at 38° increased to 11.4% when the hydrogen concentration was 0.82.

### 3.2. Hydrogen Sensor with Ag and Mg Nano-Blocks

**Figure 4:**
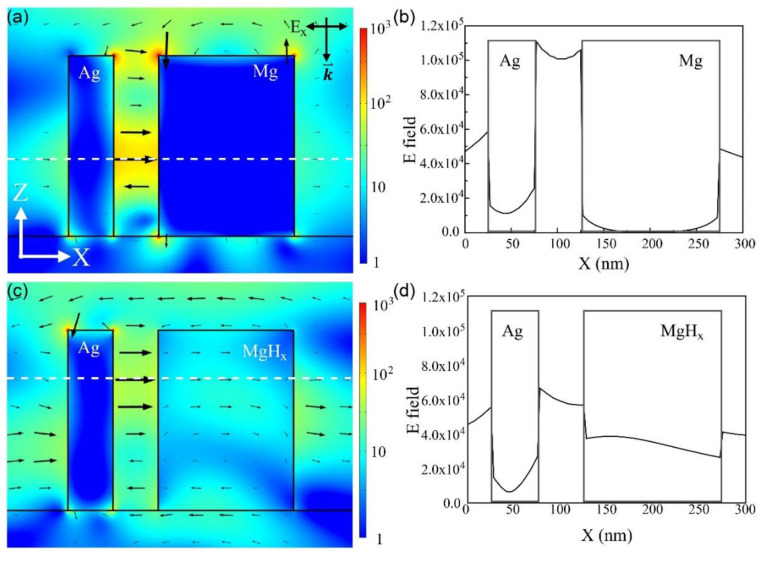
Electric (E) field intensity profiles of sensor composed of Ag and Mg nano-blocks for incident light with 400 nm wavelength: (**a**) before and (**c**) after absorbing hydrogen. (**b,d**) E field intensity along x-direction (white-dotted lines in (**a,c**) represent the z-point of maximal intensity in the gap mode between Ag and Mg blocks).

Figure 4a presents the E field intensity profile of the Ag and Mg nano-blocks before hydrogen absorption. In addition, Figure 4b shows the quantitative E field intensity at the z-point of the maximal intensity in the gap mode in the x-direction. As with the Ag and Pd nano-blocks, the E field was highly confined between the Ag and Mg nano-blocks, which means that the gap mode between the blocks was excited. When the Mg absorbed hydrogen and became MgH_x_, the E field distribution changed significantly. In contrast to the E field being unable to penetrate the metal (Ag and Mg) blocks, as seen in Figure 4a, a significant E field was observed inside the MgH_x_ block, as seen in Figure 4c, after the hydrogen absorption, because MgH_x_ is a dielectric material. According to the E field intensities along the x-direction at the maximal point of the gap modes in Figure 4b,d, the E field intensity decreased in the gap between the two blocks and increased significantly in the Mg block after the hydrogen absorption. Consequently, the effective size of the gap mode increased because the metallic Mg block became a dielectric material (MgH_x_) when absorbing hydrogen.

**Figure 5:**
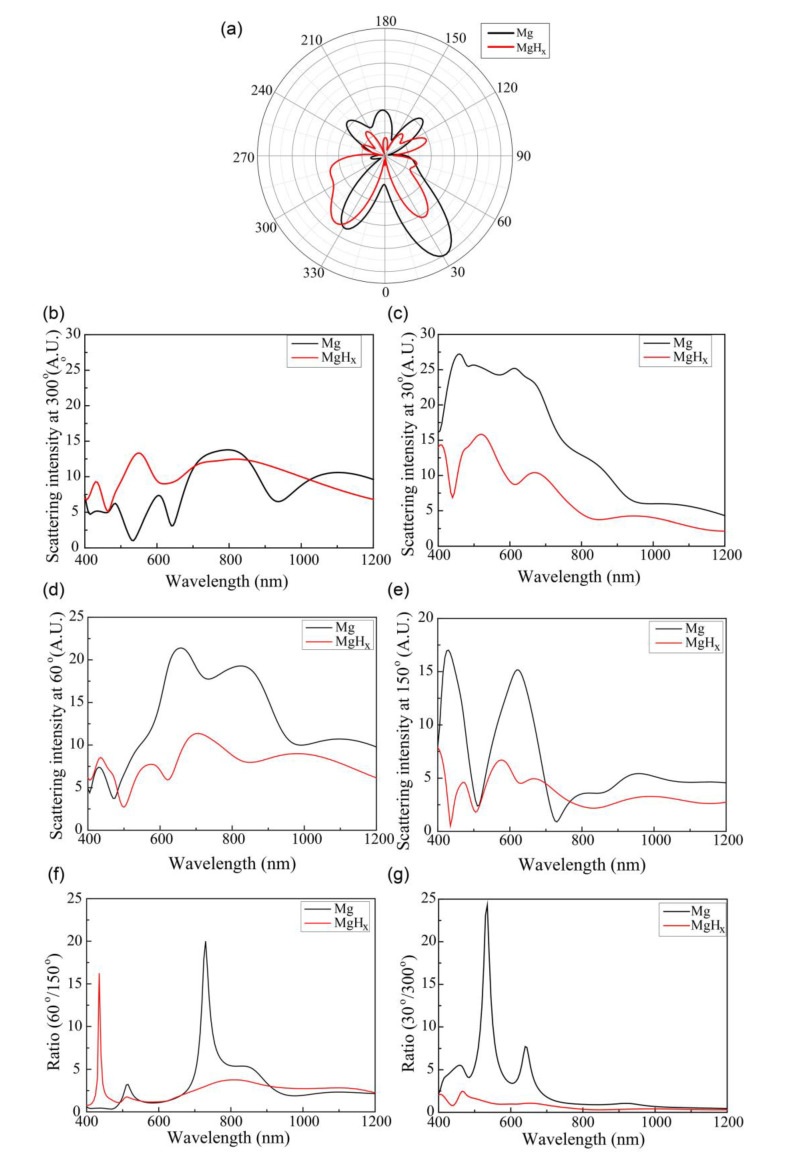
Black and red curves indicate situations before and after hydrogen absorption, respectively. (**a**) Polar graph of far-field scattering of the Ag and Mg nano-blocks for incident light with 535 nm wavelength before and after hydrogen absorption. (**b**) Far-field scattering intensity at 300° for wavelengths of 400–1200 nm (**c**) at 30°, (**d**) 60°, (**e**) and 150° before and after hydrogen absorption. (**f**) Far-field scattering intensity ratios of 60° and 150° have maxima at 730 nm (black curve) and 435 nm (red curve). (**g**) The ratio of 30° and 300° reaches a peak at 535 nm (black curve).

This great change in the E field affected the far-field distribution of the scattered light, as shown in Figure 5a. Before the Mg block absorbed hydrogen, the forward scattering intensity (black) at 0° to 90° exceeded that at 270° to 360°; however, the scattering behavior changed in the opposite direction after the hydrogen absorption (red). In other words, the far-field scattering distribution changed significantly after the hydrogen absorption. Because the metallic material Mg became the dielectric material MgH_x_ after absorbing hydrogen, the effective gap mode size broadened. Figure 5b–e show more details of the far-field scattering intensity as functions of the wavelength at 30°, 60°, 150°, and 300°. At 300° and 150°, as shown in Figure 5b,e, the far-field difference before and after the hydrogen absorption was not great for the investigated wavelength range. However, at 30°, after the hydrogen absorption, the far-field scattering intensity (red) decreased significantly over the investigated wavelength range compared with the far-field (black) scattering intensity before the hydrogen absorption, as shown in Figure 5c. At 60°, as shown in Figure 5d, the far-field scattering intensity (red) decreased such as at 30°, except in the wavelength range of 400–500 nm. Moreover, Figure 5g,f present the 30°-to-300° and 60°-to-150° scattering ratios with respect to the wavelength. As seen in Figure 5g, the ratio decreased significantly after the hydrogen absorption in the visible light range (400–700 nm). The 60°-to-150° ratio decreased the same as the 30°-to-300° ratio; however, the intensity and the range (700–800 nm) were smaller than those of the 30°-to-300° ratio. Thus, analyzing the scattering ratio of certain angles was an effective way to detect hydrogen gas. The 30°-to-300° ratio exhibited a strong peak with an amplitude of 24.31 at 535 nm before the hydrogen absorption; the ratio at 535 nm decreased to 1.19 after the hydrogen absorption. In other words, the ratio became 20.42 times lower after the hydrogen absorption for incident light of 535 nm. The 60°-to-150° ratio reached a peak of 20.01 at 730 nm, which decreased to 3.01 after the hydrogen absorption. Hence, the ratio became 6.67 times lower. Thus, analyzing the far-field 30°-to-300° scattering ratio of 535 nm wavelength light enables more sensitive detection of hydrogen gas.

### 3.3. Hydrogen Sensor Composed of Three Materials: Mg, Ag, and Pd

**Figure 6:**
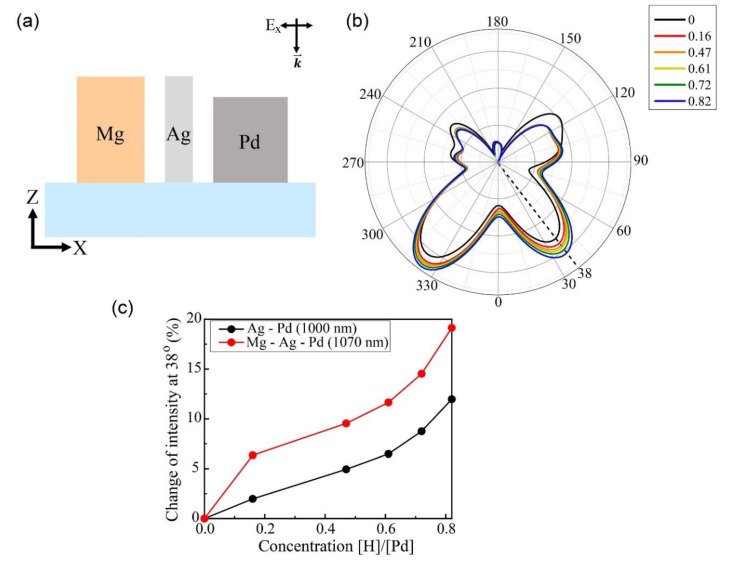
(**a**) Schematic of hydrogen sensor composed of Mg (H = 230 nm; L = 150 nm), Ag (H = 230 nm; L = 50 nm), and Pd nano-blocks (H = 200 nm; L = 150 nm) with two 50 nm air gaps. (**b**) Far-field scattering intensity with respect to hydrogen concentration (H/Pd) at 1070 nm incident wavelength. (**c**) Changes in far-field scattering intensities of two sensors at 38° with respect to hydrogen concentration (0–0.82); black curve indicates results of sensor with only Ag and Pd nano-blocks at 1000 nm wavelength; red curve indicates results of sensor with Mg, Ag, and Pd nano-blocks at 1070 nm wavelength.

As previously mentioned, the unique hydrogen absorption properties of Mg and Pd can be applied to detect hydrogen gas more sensitively. We proposed a hydrogen sensor composed of an Mg nano-block on the left, an Ag nano-block in the center, and a Pd nano-block on the right, as shown in Figure 6a. The incident light was linearly polarized along the x-direction and propagated in the negative z-direction. Figure 6b presents the far-field scattering intensity of this sensor with respect to the hydrogen concentration. The material parameters of Mg and Pd were as described in Section 2. At 38°, the far-field intensity increased monotonically with increasing hydrogen concentration. Figure 6c presents the changes in the far-field intensity at 38° of two sensors composed of different materials: Ag and Pd nano-blocks (black) and Mg, Ag, and Pd nano-blocks (red). The far-field intensity at 1000 nm wavelength monotonically increased up to 11.9% for Pd and up to 19.14% at 1070 nm wavelength for Mg and Pd with an increase of the hydrogen concentration. In other words, the change in the far-field scattering intensity of the sensor composed of Mg, Ag, and Pd blocks was greater than that of the sensor composed of Ag and Pd blocks (Figure 2) owing to the phase transition of the Mg metal block into an MgH_x_ dielectric block. In conclusion, a hydrogen sensor composed of three materials (Mg, Ag, and Pd) could sense the hydrogen concentration most effectively at 38° in the far-field scattering detection.

## 4. Conclusions

In summary, we studied three hydrogen sensing methods by analyzing the spectrum of the scattered light from the plasmonic mode of metal nano-blocks with respect to the angles and incident wavelengths. The first sensor comprised Ag and Pd nano-blocks; the Pd experienced atomic lattice expansion when it absorbed hydrogen gas. This lattice expansion affected the plasmonic gap mode between the blocks, which changed the angular scattering distribution. At a specific angle (38°) and wavelength (1000 nm), the far-field scattering intensity of the proposed sensor monotonically changed as a function of the hydrogen concentration. The second sensor exploited the unique property of Mg, which undergoes a phase transition to a dielectric material after absorbing hydrogen. The far-field scattering distribution of this sensor changed significantly after the hydrogen absorption. To achieve a better performance, the third sensor comprised Ag, Pd and Mg nano-blocks. By utilizing these materials, the hydrogen sensing performance according to concentration could be highly increased.

By using the proposed sensor, hydrogen gas concentrations from 0 to 8037 Pa could be estimated within approximately 2–5 s simply by detecting the scattering intensity at a specific angle (38°) with a photodetector with a targeted spectral bandpass filter. The proposed hydrogen sensor had advantages over the other hydrogen optical sensors. First, the sensor did not require a bulky external spectrometer, which is usually used in resonance-type plasmonic sensors; it required only a simple photodetector. Second, other plasmonic resonance-type optical sensors that detect resonance shifts had a limited spectral resolution, owing to the finite resonance linewidth. However, the proposed sensor detected changes in the far-field scattering intensity, which overcame this resolution limitation. In addition, the sensor response was faster than those of other sensor types, owing to the nano-scale physical dimension. This is because unlike bulk Pd and Mg, Pd and Mg nano-blocks only took a few seconds to absorb the hydrogen [27,28,29,30].

According to the measurements of the scattering intensity at a certain angle, this proposed nano-block plasmonic sensor has great application potential in many other optical sensor fields beyond the spectral resolution limitation. The most promising field is the measurement of the nano-position or length changes of nanostructures, or the determination of material properties by using the extremely sensitive far-field scattering spectrum of the plasmonic gap mode between two or three metal nano-blocks [9]. In addition, small changes in the strain of the substrate material and the refractive index of the environment material can be detected by using the method of the proposed sensor.

## Figures and Tables

**Table 1:** Optical properties and lattice expansion of palladium (Pd) with respect to hydrogen concentration [10,11,12].

Hydrogen Concentration H/Pd	Hydrogen Pressure (Pa)	Lattice Expansion (%)	Plasma Frequency (eV)	Relaxation Time × 10^15^ (s)	$\varepsilon_{\infty }$
0.00	0.0	0.00	7.33	2.10	1.10
0.16	467	0.818	7.30	2.07	0.80
0.47	733	2.12	7.30	2.07	0.80
0.61	1346	2.68	7.26	2.04	0.75
0.72	4185	3.12	7.11	2.04	0.75
0.82	8037	3.57	6.91	2.12	0.85
